# Retinoblastoma: Review and new insights

**DOI:** 10.3389/fonc.2022.963780

**Published:** 2022-11-02

**Authors:** Claudia Carolina Cruz-Gálvez, Juan Carlos Ordaz-Favila, Víctor Manuel Villar-Calvo, Martha Edith Cancino-Marentes, Vanessa Bosch-Canto

**Affiliations:** ^1^ Physiology Department, Centro Universitario de Ciencias de la Salud, Universidad de Guadalajara, Guadalajara, Mexico; ^2^ Pediatric Ophthalmology, Instituto Nacional de Pediatría, Universidad Autónoma de México, México City, Mexico; ^3^ Private practice, Pediatric Ophthalmology, Guadalajara, Mexico; ^4^ Master of Public Health, Universidad Autónoma de Nayarit, Tepic, Mexico

**Keywords:** retinoblastoma, intraocular tumor, leukocoria, children, ocular oncology

## Abstract

Retinoblastoma (Rb), the most frequent malignant intraocular tumor in childhood, is caused by mutations in the retinoblastoma gene (*RB1*) situated on chromosome 13q14.2. The incidence of retinoblastoma is approximately 1 in 17,000 live births with approximately 8,000 new cases diagnosed each year worldwide. Rb is the prototypical hereditary cancer in humans. Autosomal dominant inheritance is seen in 30-40% of cases whereas the non-inherited sporadic type accounts for the remaining 60-70%. Rb arises due to inactivation of both alleles of the *Rb* tumor suppressor gene, which results in a defective Rb protein (pRB) with subsequent cell cycle impairment and uncontrolled cell proliferation. Patients with Rb have survival rates higher than 95-98% in industrialized countries but mortality remains high in developing countries. For example, the mortality rate in Africa is 70%. In all cases of intraocular and extraocular retinoblastoma, there is a need for new therapies that are more effective and carry less risk of toxicity. The Bruckner test is a practical and easy test for the detection of Rb, this test consists of assessing the fundus reflex through the pupil (red reflex) in both eyes simultaneously with a bright coaxial light produced with the direct ophthalmoscope. Rb can be detected by the Bruckner test showing a pupil that shines white or “Leukocoria”. Although the diagnosis of Rb remains essentially clinical, the newly identified biomarkers could contribute to early molecular detection, timely detection of micrometastases and establish new therapeutic options for Rb.

## Introduction

**Figure 1:**
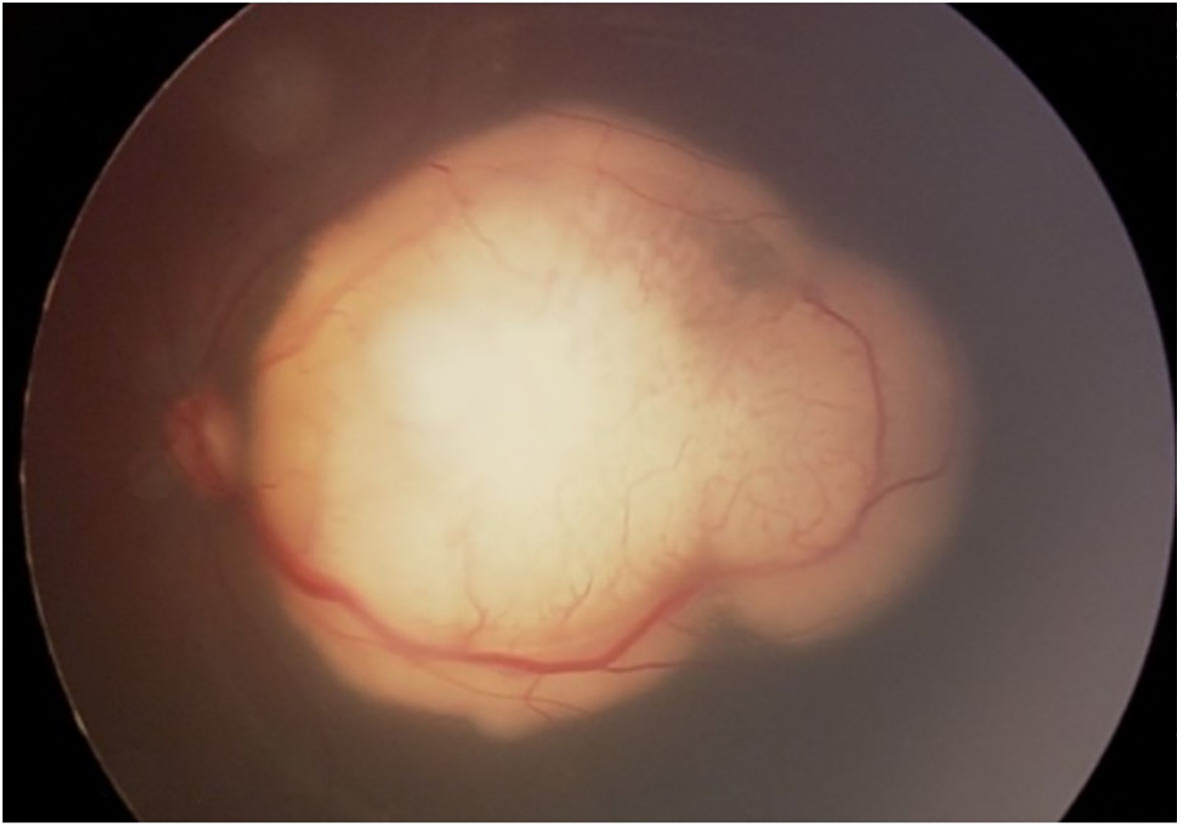
Retinoblastoma in the posterior pole.

Retinoblastoma (Rb), the most frequent malignant intraocular tumor in childhood (**Figure 1**), is caused by mutations in the retinoblastoma gene (*RB1*) situated on chromosome 13q14.2 (1, 2). The incidence of retinoblastoma is approximately 1 in 17,000 live births (3) with approximately 8,000 new cases diagnosed each year worldwide (3).

Rb is the prototypical hereditary cancer in humans. Autosomal dominant inheritance is seen in 30-40% of cases whereas the non-inherited sporadic type accounts for the remaining 60-70% (4). Rb arises due to inactivation of both alleles of the *Rb* tumor suppressor gene, which results in a defective Rb protein (pRb) with subsequent cell cycle impairment and uncontrolled cell proliferation (4).

The *RB1* gene consists of 27 exons that span 183 kilobases (kb) of genomic DNA (4) and encodes a 928 amino-acid nuclear phosphoprotein, pRb (5). pRb is a ubiquitous cell cycle regulator whose activity depends on the level of phosphorylation (5).

The hypophosphorylated form pRb arrests the cell cycle at the G1 restriction point by binding E2F transcription factors (5), which are essential for the expression of genes involved in cell cycle continuity. In Rb, the pRb is functionally inactive due to mutations or deletions (6).

pRb defective or silenced by oncoproteins produced by tumor-causing viruses (SV40, adenovirus, human papillomavirus) prevents exit from the cell cycle and apoptosis and ultimately results in uncontrolled cell division, a hallmark of cancer (1).

## Genomic changes in retinoblastoma

Amplification of the *MYCN* oncogene might initiate Rb even in absence of *RB1* mutations. These unilateral RB1(+/+) MYCN (A) tumors are characterized by distinct histological features and a very early age at diagnosis (7). Other recurring genomic changes that occur in a small minority of tumors include *BCOR* mutation/deletion and *OTX2* amplification (8).

## p53 pathway and retinoblastoma

The Rb surveillance pathway mediated by Arf, MDM2, MDMX, and p53 proteins is activated after loss of *RB1* during retinogenesis (8). Subsequent amplification of the *MDMX* gene and increased expression of MDMX protein are strongly selected during tumor progression as a mechanism to suppress the p53 response in *RB1*-deficient retinal cells. The p53 pathway is inactivated in Rb, this cancer does not originate from intrinsically death-resistant cells, as previously thought (9).

## Clinical features of retinoblastoma

The most common signs of Rb are leukocoria and strabismus (1). Leukocoria is the presenting sign in 60% of cases (2). The initial presentation is an alteration in the red reflex that, unfortunately, goes unnoticed. So, the diagnosis is usually made in advanced stages and is associated with a worse prognosis. The second most common early sign of retinoblastoma is strabismus, generally related to a macular tumor (2). Less common clinical presentations may also be observed, generally indicating advanced forms (buphthalmos, neovascular glaucoma or orbital inflammation) (2).

## Tumor patterns

### Typical tumor patterns

- Endophytic: tumor grows into the vitreous and reflects cell proliferation of the internal retinal layers (10).
- Exophytic: tumor develops beneath the retina in the subretinal space and causes overlying retinal detachment (10).
- Diffuse infiltrating: retinoblastoma develops in a flat pattern on the surface of, or beneath, the retina, with no obvious mass, no calcifications, and slow progress (10).

### Atypical tumor variants

Cavitary retinoblastoma: tumor contains cavitary spaces, loss of subretinal fluid, and seeding (4).

Anterior retinoblastoma: tumor involves the anterior chamber (4). For some authors, this form of presentation corresponds to the diffuse infiltrating pattern, where a small primary tumor arises in the peripheral retina and then seeds the anterior chamber *via* the aqueous (1, 10).

Retinocytoma: this benign variant may clinically appear as regressed retinoblastoma with associated calcification (1).

## Classification of retinoblastoma

The International Intraocular Retinoblastoma Classification (IIRC) is the current guide for Rb staging. Itwas developed by a team of retinoblastoma experts in Paris in 2003 (4, 11).

### The IIRC has 5 categories

Group A: Small tumors, 3mm or smaller in their greatest dimension, confined the retina. Located > 3 mm from the fovea and 1.5 mm from the optic disc.

Group B: Tumors greater than 3 mm, located 3 mm or less from the fovea and less than 1.5 mm from the optic disc or that presents subretinal fluid whose diameter is less than 3 mm from the margin of the tumor.

Group C: Retinoblastoma with seeding, which can be subretinal within 3 mm of the primary tumor, vitreous seeding located < 3 mm from the primary tumor, or both vitreous and subretinal seeding < 3 mm from the primary tumor.

Group D: Retinoblastoma with diffuse seeding that may be subretinal > 3 mm from the retinoblastoma, vitreous seeding >3 mm from the retinoblastoma, or a combination of both.

Group E: Extensive retinoblastoma, which occupies more than 50% of the eye socket. It can be accompanied by neovascular glaucoma, phthisis bulbi, and/or opaque media due to hemorrhage from the anterior chamber, the vitreous or the subretinal space. Tumors with post-laminar invasion of the optic nerve, choroid, sclera, orbit, or anterior chamber also enter this section.

## Trilateral and quadrilateral retinoblastoma

Trilateral retinoblastoma refers to the association of bilateral retinoblastoma with an asynchronous intracranial tumor, which occurs in fewer than 10% of bilateral cases. These tumors often arise in the pineal gland and are Primitive Neuro-Ectodermal Tumors (PNET) (pineoblastomas), but in 20-25% of cases the tumors are supra- or para-sellar (12). Rare cases of quadrilateral retinoblastoma have been reported, in which bilateral retinoblastoma is associated with both pineal-region and suprasellar intracranial primary PNET (12).

## Metastatic disease in retinoblastoma

Metastatic disease occurs in 10-15% of patients and usually in association with distinct intraocular histologic features such as deep choroidal and scleral invasion, or with involvement of ciliary body, or optic nerve beyond the lamina cribrosa (12). The key to staging patients at high risk of micrometastases will probably be based in the future on circulating biomarkers in blood. One possibility is the detection of microRNAs (2).

## Treatment of retinoblastoma

Rb, if untreated, can lead to death within 1–2 years (10) but, with adequate treatment, survival is better than 95% in developed countries (4, 13). Management of a child with retinoblastoma involves a balance of the patient’s life with globe salvage and ultimate visual potential (11).

Enucleation (with long section, 10–15 mm, of the optic nerve) alone is curative for 85–90% of children with unilateral Rb (nonheritable) and no extraocular disease (1, 11).

Conservative treatments for Rb include intravenous chemotherapy, Transpupillary ThermoTherapy (TTT), Laser Photocoagulation (LP), CryoTherapy (CT), plaque brachytherapy (ruthenium), external-beam radiotherapy (used in cases of progressive or recurrent intraocular retinoblastoma and extraocular retinoblastoma) and local chemotherapy delivered by subconjunctival (Carboplatin), subtenon (Carboplatin), intravitreal (Melphalan and Topotecan), or intra-arterial (Melphalan alone or combined with Topotecan) routes.

Intravenous chemotherapy is indicated in patients with bilateral (heritable) Rb, extraocular disease, intraocular disease with high-risk histologic features after enucleation, and intraocular disease in conjunction with aggressive focal therapies for ocular preservation (11).

Anti-retinoblastoma drugs include platinum compounds (carboplatin), etoposide, cyclophosphamide, doxorubicin, vincristine, and ifosfamide (11). Vincristine, carboplatin, and etoposide comprise the most frequently used combination (4).

Carboplatin is the basis of the intravenous chemotherapeutic scheme because the high levels attained in cerebrospinal fluid and vitreous humor (4).

High-risk retinoblastoma leads to metastasis in 24% of patients if not treated with systemic chemotherapy compared with 4% of those who receive it (11).

Management of retinoblastoma is a practiced art that involves tumor recognition, decision-making regarding the appropriate therapeutic approach, and meticulous follow-up for detection of tumor recurrence (11).

## Second malignancies after retinoblastoma

Survivors of hereditary Rb have an increased risk for developing a subsequent malignant neoplasm, for example sarcoma or melanoma (14). Treatment with External-Beam RadioTherapy (EBRT) further amplifies this risk which is heavily dependent on the age of EBRT administration (11, 15). This risk may be acceptably small for patients older than 12 months. The cumulative risk is roughly 1% per year, reaching 50% at 50 years (16). Irradiated patients have an increased risk of soft tissue sarcomas, especially leiomyosarcomas (17). Osteosarcoma is the most common tumor outside of the irradiated field (1).

## Differential diagnosis of retinoblastoma

The differential diagnoses of Rb include Coats disease, Persistent Hyperplastic Primary Vitreous, cataract, vitreous hemorrhage, ocular toxocariasis and retinal detachment (2, 18). Other possible differential diagnoses comprise intraocular inflammation, retinal detachment secondary to retinopathy of prematurity, X-linked retinoschisis, meduloepithelioma, and Norrie disease (4) (**Table 1**).

**Table 1:** Differential diagnosis of Retinoblastoma.

**Coats disease**
**Persistent Hyperplastic Primary Vitreous**
**Cataract**
**Vitreous hemorrhage**
**Ocular toxocariasis**
**Retinal detachment**
**Intraocular inflammation**
**Meduloepithelioma**
**Norrie disease**

## New treatment and diagnostic perspectives of retinoblastoma

Rb is a challenging disease. Chemotherapy has been shown to have limitations during clinical practice, mainly because of the ability of Rb to become resistant to the treatment (19). So, alternative options should be available because generation of drug resistance is a factor that contributes to the failure of chemotherapy (20).

Matrix MetaloProteinase (MMP)-2 and MMP-9 possess activity against Rb at several checkpoints that are deregulated in cancer and therefore could be adjuvant therapy in patients with Rb (21).

Promising compounds for the management of Rb have been identified in preliminary phases of drug development including inhibitors of survivin, antiapoptotic Bcl-2 family proteins, methyltransferase, and kinesin proteins (22).

New treatment modalities, namely, targeted therapies, immunotherapy, and oncolytic viruses are emerging as possible non-chemotherapeutic options in Rb (23).

Pentoxifylline is a xanthine and a non-specific phosphodiesterase (PDE) inhibitor that inhibits the phosphorylation of I kappa B-alpha (IĸBα) in serines 32 and 36, and this disrupts NF-ĸB activity. Pentoxifylline in combination with different antitumoral drugs increases the levels of apoptosis *in vivo* and *in vitro* studies and can induce increasing apoptosis in children with acute lymphoblastic leukemia (24–34). Pentoxifylline with carboplatin combination exhibited a high rate of apoptosis in human Y79 retinoblastoma cells. These findings suggest that the combination of pentoxifylline with carboplatin may comprise a promising strategy for the treatment of Rb (35).

Epigenetics is widely recognized to play a fundamental role in ocular pathologies (36). Rb tumorigenesis and progression require additional genetic and epigenetic alterations following *RB1* inactivation (37). Epigenetic dysregulation in Rb has been observed for nearly all areas of epigenetics including DNA methylation, histone modifications, and noncoding RNAs as exemplified by promoter hypermethylation of tumor suppressor genes, activating histone modifications at the promoter of cancer pathway genes such as SYK, and aberrant regulation of microRNAs (miRNA´s) (37), and Circular RNAs (circRNAs) (38).

Long noncoding RNAs (lncRNAs) are defined as RNA transcripts longer than 200 nucleotides that have no protein-coding ability (39). LncRNA-UCA1 could promote cell proliferation and cell cycle progression and inhibit cell apoptosis in Rb by activating the PI3K/Akt pathway (39). LncRNA TUG1 has also been recognized as an oncogene in several cancers (40). TUG1 was upregulated in Rb cells and the absence of TUG1 repressed cell proliferation whereas it accelerated cell apoptosis in Rb. In brief, TUG1 is an oncogenic gene in Rb (40).

MicroRNA (miRNA) is one class of small non-coding RNA (sncRNA) that participates in a variety of biological process *via* the targeting sequence of cellular and molecular pathways (41). The oncogenic microRNA miR-17-92 has been implicated in Rb tumorigenesis (42). Expression of miR-17-92 induces rapid proliferation and disease onset. This increase in proliferation is linked to the miR-17 sub-family, which targets cell-cycle inhibitors p21 and p57 (43). miR-204 acts as a tumor suppressor, while it has much less expression in patients with retinoblastoma (44). Cyclin-D2 and MMP-9 are two key genes that are regulated by miR-204 in retinoblastoma. High expression of cyclin-D2 and MMP-9 increases the cell division rate and progression of RB (44). miR-17-3P, miR-17-5P, miR-18a, and miR-20a are highly expressed in the serum of children with Rb (45). Cone-rod homebox (CRX) and Otx-like homebox transcriptor for photoreceptor transcription have been reported as potential biomarkers in Rb (2). CTX messenger RNA is also a promising marker for the detection of micrometastases (2). miRNAs could be used as reliable biomarker for the diagnosis of RB or will be able to predict the risk of micrometastases, in the fairly near future (2, 45).

Circular RNAs (circRNAs) have vital roles in human cancers, including retinoblastoma (RB) (38). Circ-FAM158A knockdown inhibits retinoblastoma cell proliferation, metastasis and promotes apoptosis *in vitro* and *in vivo* (38). Circ_0075804 promotes RB progression through miR-138-5p-dependent regulation of PEG10 (46). CircMKLN1 overexpression slows RB progression through miR-425-5p spongylation and PDCD4 upregulation (47) and silencing circ-E2F3 inhibits proliferation, migration and invasion, and induces apoptosis of retinoblastoma cells *in vitro*, as well as reduces retinoblastoma growth *in vivo* (48). These findings could represent potential effective targets for the treatment of retinoblastoma.

Copy number alterations (CNA) have been identified and translated to current clinical practice for retinoblastoma (49). CAN reported for intraocular retinoblastoma are 1q, 2p, 6p, and 17q gains and 16q, 11q, 19q and 21q losses (49). A small percentage of patients present recurrent somatic mutations in BCL6 Corepressor gene (BCOR) (49). Analysis by Aschero et al. of CNA and BCOR gene alterations show that CNA previously reported for intraocular retinoblastoma were also found in extraocular retinoblastoma: gains in 1q, 2p, 6p, 17q and losses in 16q, 19q and 11q, in addition to BCOR alterations (49). In metastatic retinoblastoma cases included analysis of genes associated to gains in 1q (including MDM4, KIF14 genes), 2p (MYCN), and 6p (DEK, E2F3) and 16q (CDH11) deletion (49). The ATM tumor suppressor gene was significantly altered in cases with 11q deletion (49).

Clear corneal paracentesis is part of the standard intravitreal chemotherapy injection protocol (50). Kim et al. propose the extraction of the aqueous humor (AH) to be used as a liquid biopsy, or surrogate to tumor biopsy, for retinoblastoma. The safety method described by Kim et al. establishes the needles can only enter the anterior chamber and should not make contact with the iris or lens. It is most important that the needle never enters the vitreous cavity (unless combined with chemotherapy delivery), or contacts the tumor as this hypothetically elevates the risk of tumor seeding and extraocular extension of disease (50). Although clinical validity of the AH liquid biopsy platform for RB has been established, it is currently approved for research only; the AH liquid biopsy has the potential to enable precision oncology in the future, for RB (50).

Cheng and collaborators found the concentrations of IL-6, IL-7, IL-8, IFN-γ, PIGF-1, VEGF-A, β-NGF, HGF, EGF, and FGF-2 were significantly higher in the Aqueous Humor (AH) of patients with Rb than in those in the control group. These findings could contribute to the implementation of novel strategies for the diagnosis and therapy of Rb (51).

For children diagnosed with Rb, the dysregulation of methylation in *MSH6, CD44, PAX5, GATA5, TP53, VHL, GSTP1, MGMT, RB1*, and *CDKN2* genes is a further tool for targeted treatment to improve the prognosis for this ocular cancer (52).

## Discussion

Patients with Rb have survival rates higher than 95-98% in industrialized countries but mortality remains high in developing countries (2, 4, 12). For example, the mortality rate in Africa is 70% (44).

In all cases of intraocular and extraocular retinoblastoma, there is a need for new therapies that are more effective and carry less risk of toxicity (23).

Epigenetic studies have shown that changes in the epigenome contribute to the rapid progression of retinoblastoma following classic genetic changes. The targetable nature of epigenetic modifications provides a unique opportunity to optimize treatment paradigms and establish new therapeutic options for retinoblastoma with these aberrant epigenetic modifications (53).

The identification of the biomarkers and chromosomal copy number alterations described in this article could help guide future clinical management of Rb patients, for example could correlate with a more aggressive tumor (49).

Not only does the AH liquid biopsy provide the opportunity to better understand intratumoral dynamics in eyes that are actively undergoing therapy, but it also has the potential to improve patient care in the future (50).

The use of cancer screening modalities has been suggested for the goal of minimizing morbidity and mortality in the pediatric population. For example, the Bruckner test is a practical and easy test for the detection of Rb, this test consists of assessing the fundus reflex through the pupil (red reflex) in both eyes simultaneously with a bright coaxial light produced with the direct ophthalmoscope (54–65). Retinoblastoma can be detected by the Bruckner test showing a pupil that shines white or “Leukocoria”. In early stages, the Rb can be detected through a minimal alteration in the Bruckner test. Early and timely diagnosis of Rb can be life-saving.

All physicians (General Practioners or Family physicians, Pediatricians, Neonatologists, Ophthalmologists, and Pediatric Ophthalmologists) who have contact with children should perform the Brucker test in order to achieve a timely detection of Rb.

## Conclusion

Retinoblastoma, the most frequent malignant intraocular tumor in childhood, is caused by mutations in the retinoblastoma gene (*RB1*). Although the diagnosis of Rb remains essentially clinical, the newly identified biomarkers could contribute to early molecular detection, timely detection of micrometastases and establish new therapeutic options for Rb.

## Author contributions

VMV-C, VB-C, and CCC-G contributed to conception and design of the study. CCC-G and MEC-M organized the database. VB-C, MEC-M, and CCC-G wrote the first draft of the manuscript. JCO-F, VMV-C, VB-C, and CCC-G wrote sections of the manuscript. All authors contributed to manuscript revision, read, and approved the submitted version.

## Conflict of interest

The authors declare that the research was conducted in the absence of any commercial or financial relationships that could be construed as a potential conflict of interest.

## Publisher’s note

All claims expressed in this article are solely those of the authors and do not necessarily represent those of their affiliated organizations, or those of the publisher, the editors and the reviewers. Any product that may be evaluated in this article, or claim that may be made by its manufacturer, is not guaranteed or endorsed by the publisher.
